# Manual of Materia Medica and Pharmacy

**Published:** 1904-09

**Authors:** 


### Bibliography.

Manual of Materia Medica and Pharmacy. Specially designed for the use of Practitioners and Medical, Pharmaceutical, Dental, and Veterinary Students. By E. Stanton Muir, Ph.G., V.M.D., Instructor in Comparative Materia Medica and Pharmacy in the University of Pennsylvania. Third Edition. F. A. Davis Company, Philadelphia, 1904.

The first edition of this book was published eight years ago. The object, as stated in the preface, is, "To give to practitioners and students of medicine, in as concise and clear a manner as possible, those points which are of value, without the lengthy detail usually found in text-books."

There is always a danger in this condensing, inasmuch as matter vital to a correct understanding of the character of the drug and its therapeutic use may be practically eliminated. It is a question whether a student can study materia medica through a mere outline of the drugs described, any more than the student of anatomy could confine himself to the skeleton to acquire a knowledge of the human organism in its entirety. The true student will demand, and earnestly seek, other and fuller sources of information. While this defect is apparent in this book, it is not a subject for extended criticism in this respect.

The book will be valuable to dental students, but in a limited degree, as it fails to deal in the dental use of drugs. There is a general opinion prevalent that the therapeutic use of drugs is the same in all diseases of the human organism. While this is true the specialist finds general directions not always available. It is equally true that these special details must be taught by and received from the teacher, but a line or two in this direction would be of decided advantage to a book prepared for dental workers as well as veterinarians.

The real fault of the book is that it fails to notice some of the most used drugs in dentistry, especially of the important antiseptic class. In illustration it is only necessary to mention formalin and naphthol. It can hardly be said that these have not been sufficiently tried as to their value. It would seem that these might be found of decided value in veterinary medicine, in which the author is evidently most at home. They have been so long used in dentistry that the well-trained practitioners regard these, and others, indispensable in every-day work.

Part I. of the book is devoted to Botany, Part II. to Drugs, and Part III. to Pharmacy. This makes an excellent arrangement. The drugs follow each other in alphabetical order. This is much more satisfactory than that adopted in some of the recent works on Materia Medica. While much may be said in favor of other methods of arranging the drugs, it is thought that the method adopted by the author is the best for the average student.

The use of interleaved blanks for memoranda is an excellent aid, and is worthy of adoption in more pretentious publications. Aside from the criticisms made, the book, as a whole, must be regarded as superior to the ordinary manuals and of distinct value as an aid to the study of the subject.

